# Artificial Intelligence-Based Triage and Diagnostic Support for Gastric Biopsies: A Multi-Centre Prospective Evaluation Across NHS Wales

**DOI:** 10.3390/diagnostics16213553

**Published:** 2026-10-28

**Authors:** Muhammad Aslam, Angela Phillips, Pearl Huey, Namor Williams, Andrew Powell, Vincent Onyekwelu, Dyfrig Mason, Maya Grinwald, Rachel Mikulinsky, Giuseppe Mallel, Simone Ambrogio, Chaim Linhart, Manuela Vecsler

**Affiliations:** 1 Betsi Cadwaladr University Health Board, Bangor LL57 2PW, Wales, UK; muhammad.aslam3@wales.nhs.uk (M.A.); angela.phillips2@wales.nhs.uk (A.P.); pearl.huey@wales.nhs.uk (P.H.); 2 Swansea Bay University Health Board, Swansea SA12 7BR, Wales, UK; namorwyn.williams@wales.nhs.uk (N.W.); andrew.powell@wales.nhs.uk (A.P.); 3 Hywel Dda University Health Board, Carmarthen SA31 3BT, Wales, UK; vincent.onyekwelu@wales.nhs.uk (V.O.); dyfrig.mason@wales.nhs.uk (D.M.); 4 Ibex Medical Analytics, Tel Aviv 6902068, Israel; maya.grinwald@ibex-ai.com (M.G.); rachel.mikulinsky@ibex-ai.com (R.M.); giuseppe.mallel@ibex-ai.com (G.M.); simone.ambrogio@ibex-ai.com (S.A.); chaim.linhart@ibex-ai.com (C.L.)

**Keywords:** artificial intelligence, digital pathology, gastric cancer, clinical triage, workflow optimisation, diagnostic support, early diagnosis, turnaround time, NHS Wales

## Abstract

**Background**: Histopathology workforce shortages and operational backlogs severely delay gastric cancer reporting. This multi-centre prospective evaluation assessed the diagnostic accuracy, clinical safety-net utility, and workflow triaging efficiency of an artificial intelligence (AI) platform for gastric biopsies across NHS Wales. **Methods**: A prospective cohort of 848 consecutive gastric biopsy cases across three NHS Health Boards was processed using a commercial AI platform. Diagnostic performance was evaluated against standard-of-care clinical reference diagnoses. Post-analytic cross-tabulation simulated dynamic AI-driven worklist prioritisation to quantify triage safety, false-positive alerts, and turnaround time impact across urgent and routine pathways. **Results**: For gastric malignancies (*n* = 40), the AI platform achieved a sensitivity of 100.0% (95% CI: 91.2–100.0%; AUC 0.986) and a specificity of 97.2% (95% CI: 95.8–98.1%). Sensitivities for low-grade dysplasia/adenoma (*n* = 12) and gastric intestinal metaplasia (*n* = 98) were 100.0% and 94.9%, respectively. The AI review functioned as a parallel safety net, capturing eight primary lesions missed during routine human evaluation, including one occult adenocarcinoma. In workflow simulation, the platform correctly prioritised 100.0% of cancers, including 6 cancers hidden within routine backlogs, and 97.2% (106/109) of high-risk non-cancer precursors, with false-positive alerts in 4.6% (39/848) of all cases. **Conclusions**: AI-assisted gastric pathology review demonstrates exceptional diagnostic accuracy, acts as a reliable clinical support tool, and enables effective risk-stratified triage. Deploying AI triage in public healthcare infrastructure offers a scalable solution with the potential to reduce diagnostic backlogs and accelerate treatment for high-risk oncology patients.

## 1. Introduction

Gastric cancer remains a significant clinical challenge, largely because it is frequently diagnosed at advanced pathological stages when treatment efficacy is severely compromised and prognosis is poor. Population-level data from Cancer Research UK underscore a stark, stage-dependent survival gradient: while approximately 65% of patients diagnosed at stage 1 survive for five years or more, five-year survival rates decline steeply to 35% at stage 2, and drop to virtually 0% by stage 4, where only 20% of patients survive for a single year [1]. Beyond early cancer detection, identifying pre-malignant lesions, such as gastric intestinal metaplasia (GIM) and low-grade dysplasia (LGD), offers a vital clinical window to interrupt the gastric carcinogenesis pathway [2,3,4]. When recognised early, these lesions enable risk-stratified surveillance and interventions such as *Helicobacter pylori* eradication strategies that can halt progression or induce regression [2,3,4,5,6].

Despite clear clinical incentives for early intervention, histopathology services globally are facing unprecedented structural demands and workforce shortages, resulting in widespread diagnostic backlogs [7,8,9,10]. This universal challenge is accurately reflected within the UK National Health Service (NHS) framework, providing a highly representative environment for evaluating digital health solutions. In the standard clinical workflow, gastric biopsies are manually triaged into “Urgent” or “Routine” pathways based on initial endoscopy findings or available clinical notes. While urgent gastric specimens suspicious for malignancy are targeted for reporting within 7 to 10 working days to meet national standards [11], routine endoscopic biopsies face protracted turnaround times, averaging 15 to 30 working days in real-world practice, with operational backlogs extending reporting beyond 50 working days during peak demand. Because occult malignancies or high-risk precursors can occasionally mimic benign lesions, these operational lags may introduce clinical vulnerability across any healthcare system. Reflecting these broad systemic pressures, national metrics indicate that while 70% of urgent UK referrals meet the 28-day Faster Diagnosis Standard to confirm or rule out cancer, only 55% to 60% of oncology patients in Wales commence first definitive treatment within the statutory 62-day target [12]. Recognising these constraints, the Welsh healthcare ecosystem has positioned itself at the forefront of digital pathology innovation, serving as an ideal real-world paradigm to evaluate how AI-driven triage could optimise workflows and safeguard patient care.

To mitigate these diagnostic delays under high workloads, artificial intelligence (AI) solutions offer a promising paradigm shift [13]. Building upon established successes in other tissue domains, most notably in prostate and breast pathology, AI diagnostic tools have demonstrated transformative potential in streamlining workflows, reducing turnaround times, reducing ancillary diagnostic tests, and effectively triaging patients [14,15,16,17,18,19]. Because gastric cancer is highly time-sensitive and historically prone to severe reporting delays, translating these AI-driven triage capabilities to gastric pathology offers a vital opportunity to strengthen early detection and prevention frameworks [20,21].

Here, we present a multi-centre clinical service evaluation of the Ibex Gastric AI platform, deployed prospectively at scale across three major NHS Health Boards in Wales (BCUHB, HDUHB, and SBUHB) under the SBRI Cancer Challenge. Between March and June 2025, this project evaluated a consecutive, real-world cohort of gastric biopsy cases uploaded directly into existing “Routine” and “Urgent” workflows. The primary objective of this evaluation was to validate the clinical utility of the AI platform as a triage solution as well as a decision support solution, assessing its clinical effectiveness, safety, efficiency, and added value. By enabling early triage and the prioritisation of urgent cases based on AI insights, this workflow simulation evaluated the potential of the platform to streamline workflows, shorten turnaround times, and potentially reduce waiting lists from months to days, ultimately facilitating earlier diagnosis, faster access to treatment, and improved outcomes for patients.

## 2. Materials and Methods

### 2.1. Study Design and Clinical Cohort

This multi-centre, prospective clinical service evaluation study was conducted across three major NHS Health Boards in Wales: Betsi Cadwaladr University Health Board (BCUHB), Hywel Dda University Health Board (HDUHB), and Swansea Bay University Health Board (SBUHB). Between March and June 2025, a total of 982 consecutive gastric biopsy cases were prospectively uploaded to the AI platform (Ibex Gastric AI, Ibex Medical Analytics, Tel Aviv-Yafo, Israel). At the time of upload, cases remained categorised as “Routine” or “Urgent” according to the historical clinical pathway established by each site.

To minimise disruption to the routine clinical workflow, whole-slide images of entire gastrointestinal (GI) cases were uploaded to the AI platform. This included multi-well tissue specimens containing oesophageal, duodenal, gastric, and colonic tissue, allowing the reporting pathologist to visualise the entire case within a single whole slide image (WSI) viewer.

However, the evaluation focused exclusively on gastric tissue in compliance with regulatory approvals for the AI platform under strict CE-IVDR and Medicines and Healthcare products Regulatory Agency (MHRA) regulatory certifications. To ensure a strict evaluation of gastric pathology, an exclusion protocol was enacted. Reviewing pathologists indicated a priori whether the uploaded tissue was eligible for the study by confirming if it contained gastric tissue or other non-gastric tissue types (e.g., duodenum, oesophagus).

Within this study, Leica Aperio GT 450 (Leica Biosystems GmbH, Nussloch, Germany) scanners were utilised across three sites. Slides were scanned at 40× magnification with a spatial resolution ranging from 0.20 to 0.28 microns per pixel (µm/pixel), generating whole-slide images (WSIs). The scanned WSIs were uploaded using an encrypted and secure file transfer protocol (SFTP) onto a dedicated Virtual Private Cloud (VPC) environment hosted within the UK region of Amazon Web Services (Amazon Inc., Seattle, WA, USA), ensuring strict compliance with GDPR and applicable UK data protection regulations. Data transfer and processing were managed with data encryption at rest (AES-256) and in transit (TLS 1.2, ECDHE_RSA with P-256, AES_128_GCM).

### 2.2. AI Platform

**Figure 1:**
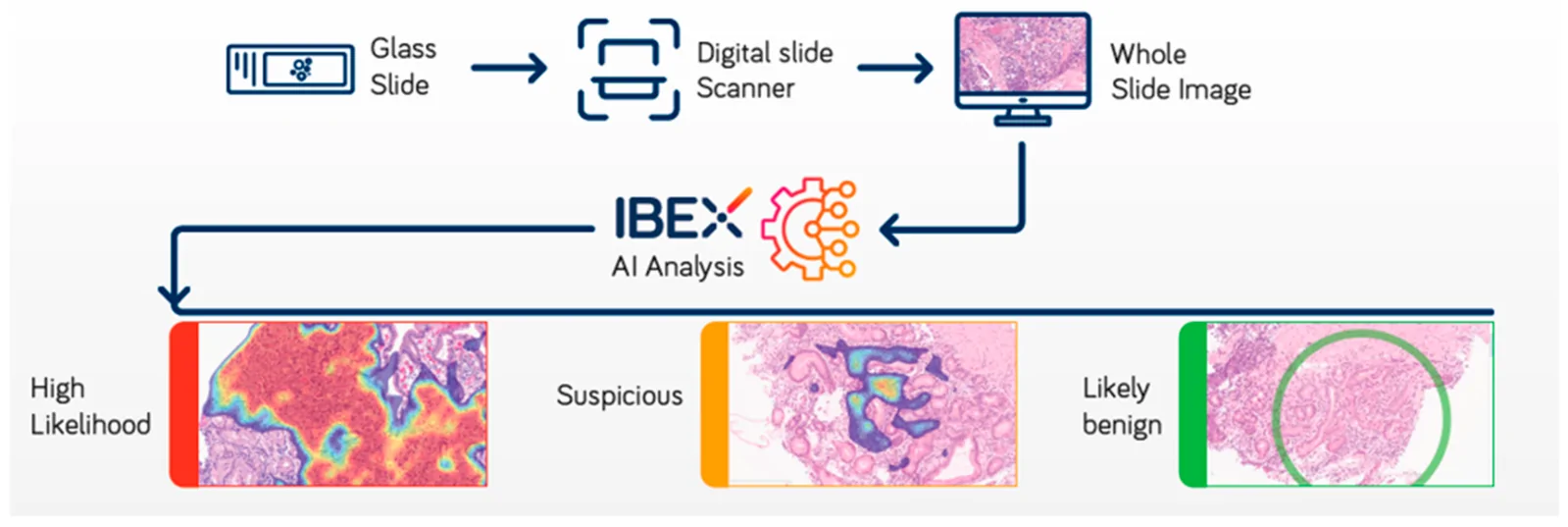
AI-based workflow with pre-screening and triage of biopsies.

The AI algorithm (Ibex Gastric, Ibex Medical Analytics, Tel Aviv-Yafo, Israel) was designed to support pathologists in primary diagnosis when reviewing gastric biopsies on WSIs. The algorithm is based on an ensemble of multilayered convolutional neural networks (CNNs) and was designed to classify and analyse a WSI in four consecutive steps: (1) tissue detection, (2) classification, (3) heatmap visualisation and (4) slide-level analysis. Tissue detection and quality control were performed at 5× magnification (96 × 96-pixel patches) using an EfficientLite convolutional neural network trained on approximately 17,600 annotated patches to distinguish tissue, blurry tissue, and background (4-fold cross-validation AUCs: 0.997, 0.978, and 0.996, respectively), automatically flagging slides unsuitable for downstream analysis [22]. Following quality filtering, tissue was classified into 34 clinically relevant groups derived from 87 histomorphological labels spanning normal, inflammatory, metaplastic, and neoplastic gastric entities. Model training leveraged 1,733,208 labelled patches derived from 4273 WSIs (40× magnification; 0.20–0.28 μm/pixel) annotated by 31 senior pathologists across 12 international laboratories using diverse scanner vendors (Philips, Leica, Hamamatsu, 3DHistech), with intentional enrichment for rare conditions and hard-to-classify morphologies to optimise performance and robustness. The high-level system workflow proceeds as follows, and it is shown in Figure 1.

- Triage Sorting: The system aggregates findings to generate a case-level and slide-level likelihood score for malignancy or specific precursor conditions. Cases may be dynamically sorted on the pathologist’s worklist according to condition severity using the “AI Findings” column, prioritising high-risk cases regardless of their original chronological or manual triage designation.
- Visualisation: Pathologists review cases through a digital slide viewer equipped with interactive cancer heatmaps that highlight suspicious regions of interest in red (“high likelihood”), yellow (“medium likelihood”), or green (“low likelihood”), while maintaining full control of the final diagnosis as per the established standard of care.

### 2.3. Pathological Target Conditions and Threshold Optimisation

In this evaluation study, the AI platform was specifically configured to identify and triage both malignant and pre-malignant conditions, grouped into distinct clinical targets:

- Cancer-Related Findings: Carcinoma, high-grade dysplasia (HGD), and high-grade lymphoma.
- Pre-Cancerous/Precursor Lesions: Low-grade dysplasia (LGD), adenoma, and gastric intestinal metaplasia (GIM).
- “Other” Pathologies: Neuroendocrine neoplasms (NEN) and low-grade lymphoma.

Before study data collection commenced, the three Health Boards provided a separate, enriched dataset comprising a total of 150 cases covering all predefined pathological gastric lesions to establish class-specific alert-triggering thresholds. The AI model was neither retrained nor modified and continued to generate standard probability scores on a scale of 0–1. The thresholds were selected to prioritise sensitivity while maintaining sufficient specificity to limit false-positive alerts. These thresholds were fixed before evaluation, with no subsequent tuning using the study cohort.

This triage-oriented approach aimed to minimise false negatives while supporting clinical workflow efficiency, acknowledging the potential trade-off with positive predictive value (PPV), particularly for low-prevalence lesions.

### 2.4. Reference Standard and Discrepancy Adjudication

Primary diagnostic reporting was performed by NHS pathologists according to routine standard-of-care (SoC) practice, using digital slides and without AI assistance. Only after completion of the primary reports were cases evaluated using the AI platform, and its outputs compared with the original SoC diagnoses. This post-analytic evaluation ensured that AI outputs did not influence the initial clinical reporting.

Where AI findings agreed with the original SoC diagnosis, the diagnosis was retained as the reference standard. Where discrepancies were identified, including suspicious findings flagged by AI but not documented in the original diagnosis, cases underwent unblinded secondary review. The final adjudicated diagnosis was established through discussion between at least two expert pathologists, with ancillary investigations, including immunohistochemistry, performed where clinically appropriate. This approach preserved routine patient care while supporting expert resolution of discrepancies and reducing reliance on AI outputs alone when establishing the final clinical reference standard.

### 2.5. Statistical Analyses

Standard diagnostic performance metrics (sensitivity, specificity, positive predictive value (PPV), negative predictive value (NPV)) were computed for the AI results across the evaluated clinical categories and compared to the established final clinical diagnosis, which served as the reference standard. Additionally, the overall discriminatory capability of the system was quantified by calculating the area under the receiver operating characteristic curve (AUC). Two-sided 95% confidence intervals (CI) for proportions (sensitivity, specificity, PPV, NPV) were calculated using the Clopper–Pearson exact binomial method, while 95% CIs for ROC-AUC were computed using DeLong’s method.

### 2.6. Historical Baseline Data and Operational Workflow Simulation

To establish a baseline for standard of care (SoC), operational turnaround times (TAT) were retrospectively extracted for consecutive gastric pathology specimens processed between January 2025 and June 2025 across three participating NHS Health Boards (Betsi Cadwaladr University Health Board, Swansea Bay University Health Board, and Hywel Dda University Health Board). Historical data were retrieved directly from the respective Laboratory Information Management Systems (LIMS), covering both the “Routine” and “Urgent” diagnostic pathways. To reduce the influence of extreme values, outliers were identified using the 1.5 × IQR rule and excluded before calculating the mean TAT, standard deviation, and median. Prior to analysis, all datasets were fully anonymised at the source by local hospital information governance teams in compliance with UK GDPR and NHS data protection standards.

To evaluate the potential impact of AI-driven worklist orchestration without altering standard-of-care patient pathways during clinical validation, a non-interventional workflow simulation was performed. Prospective AI findings were post-analytically cross-tabulated against original LIMS triage designations (‘Routine’ versus ‘Urgent’) to model an automated workflow where high-risk cases (malignancies and precursor lesions) were flagged for immediate reading queue prioritisation upon digitisation, framed against historical baseline turnaround times.

## 3. Results

### 3.1. Cohort Characteristics

The evaluation study included 982 total uploaded consecutive prospective cases across the three participating Health Boards as detailed in Table 1. Of the initial 982 cases, 134 were excluded after being identified as non-gastric tissues (e.g., gastroesophageal junction, duodenal, or colon biopsies). The final cohort comprised 848 cases. For sub-analyses of pre-malignant precursor lesions (low-grade dysplasia and intestinal metaplasia), six confirmed cancer cases were excluded (*n* = 842) because baseline diagnostic reports focused exclusively on the primary malignancy, leaving co-existing background features unrecorded in the reference data.

### 3.2. Overall Diagnostic Performance

The diagnostic performance was evaluated on a total of 848 consecutive prospective cases across the three participating Health Boards as detailed in Table 1. The AI platform demonstrated high diagnostic accuracy across all evaluated pathological categories combined, yielding an overall sensitivity of 98.5% (95% CI: 94.8–99.6%) and a specificity of 94.7% (95% CI: 92.8–96.1%). The underlying diagnostic classification counts (true positives [TP], true negatives [TN], false positives [FP], and false negatives [FN]) for the overall cohort and each participating Health Board are provided in Appendix A (Table A1, Table A2, Table A3 and Table A4).

For gastric malignancies (including carcinoma, HGD, and high-grade lymphoma) specifically (*n* = 40), the algorithm achieved an absolute sensitivity of 100.0% (95% CI: 91.2–100.0%), a specificity of 97.2% (95% CI: 95.8–98.1%), and an AUC of 0.986 (95% CI: 0.960–1.000), successfully identifying all malignant cases within the multi-centre cohort (Table 2). Across all three Health Boards, performance remained consistently high, with 100% sensitivity for cancer detection maintained at every individual site (Table A2, Table A3 and Table A4).

**Figure 2:**
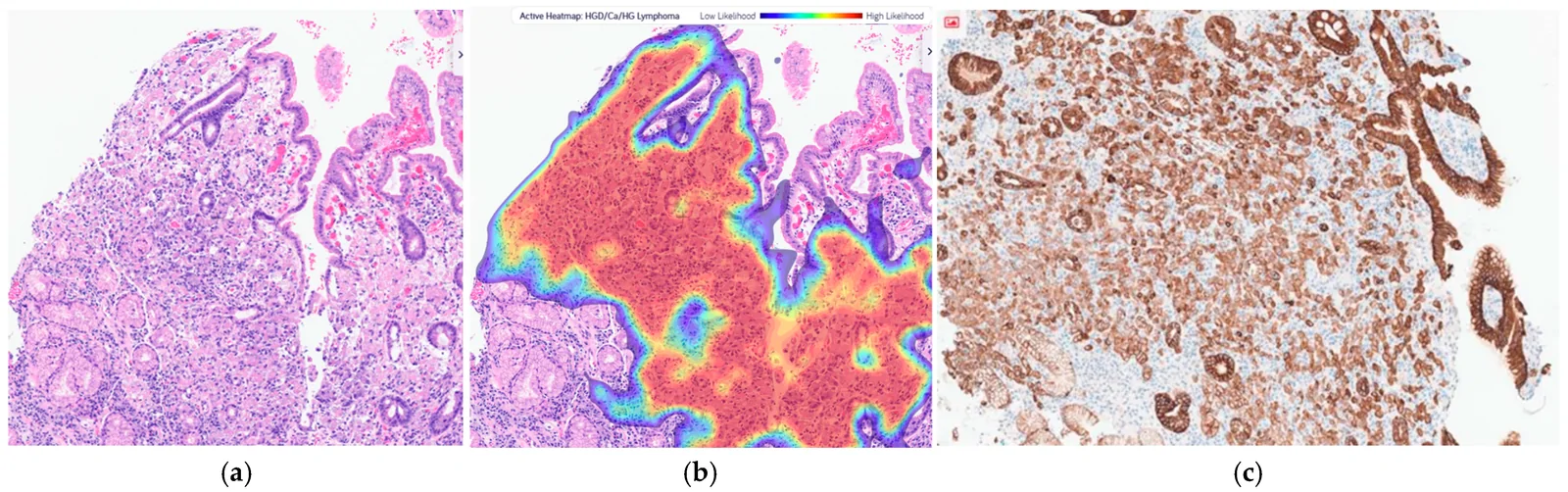
AI heatmap identifying an occult gastric adenocarcinoma originally misclassified as a benign gastric ulcer during primary standard of care reporting: (**a**) digital H&E slide at 10× magnification of the cancer area; (**b**) digital H&E slide at 10× magnification of the cancer area with overlapping AI heatmap, indicating high probability of malignancy. The active heatmap encompasses high-grade dysplasia (HGD), carcinoma and high-grade lymphoma. The colour bar indicates predicted likelihood, ranging from blue (low likelihood) to red (high likelihood); (**c**) immunohistochemistry slide at 10× illustrating cytokeratin-positive (CK+) cell infiltrates.

Crucially, parallel to the standard of care (SoC) review, the AI review functioned as a clinical safety net, capturing 8 primary lesions that were completely missed during the original standard of care diagnosis. The most critical one involved a routine-track case signed off during primary diagnostic evaluation as a benign gastric ulcer (Figure 2a). The algorithm analysis showed high probability of malignancy (Figure 2b), prompting the site’s clinical lead to re-examine the case. Subsequent reflex immunohistochemical (IHC) analysis confirmed cytokeratin-positive (CK+) cell infiltrates (Figure 2c), establishing a definitive diagnosis of gastric adenocarcinoma and shifting the patient’s trajectory to immediate oncological care.

### 3.3. Identification of Pre-Malignant Lesions

**Figure 3:**
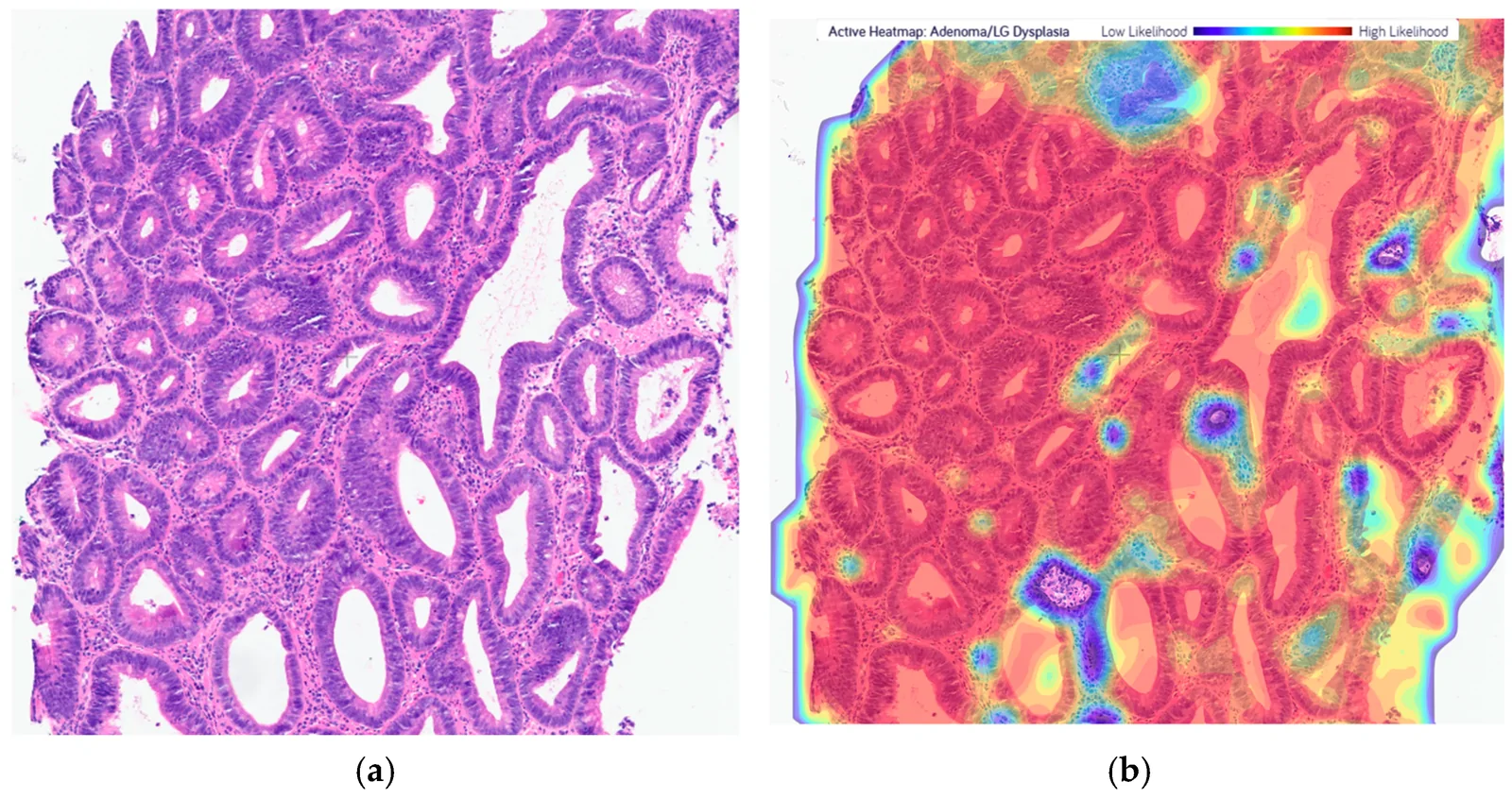
Automated detection and regional classification of low-grade gastric dysplasia: (**a**) digital H&E slide at 10× magnification of the region of interest; (**b**) digital H&E slide at 10× magnification of the region of interest with overlapping AI heatmap indicating high probability of low-grade gastric dysplasia. The active heatmap encompasses low-grade dysplasia and adenoma. The colour bar indicates predicted likelihood, ranging from blue (low likelihood) to red (high likelihood).

In the subset of 842 non-cancer cases, the platform demonstrated high accuracy in triaging the pre-malignant cases. For low-grade dysplasia (LGD) and adenoma (*n* = 12), the platform achieved a sensitivity of 100.0% (95% CI: 75.8–100.0%), a specificity of 98.0% (95% CI: 96.7–98.7%), and an AUC of 0.990 (95% CI: 0.950–1.000) (Table 2, Figure 3). The wide confidence interval for sensitivity highlights the uncertainty associated with the small number of positive cases. The recorded positive predictive value (PPV) of 41.4% directly reflects a low baseline disease prevalence in this prospective sample alongside a software configuration deliberately optimised to prevent false negatives.

**Figure 4:**
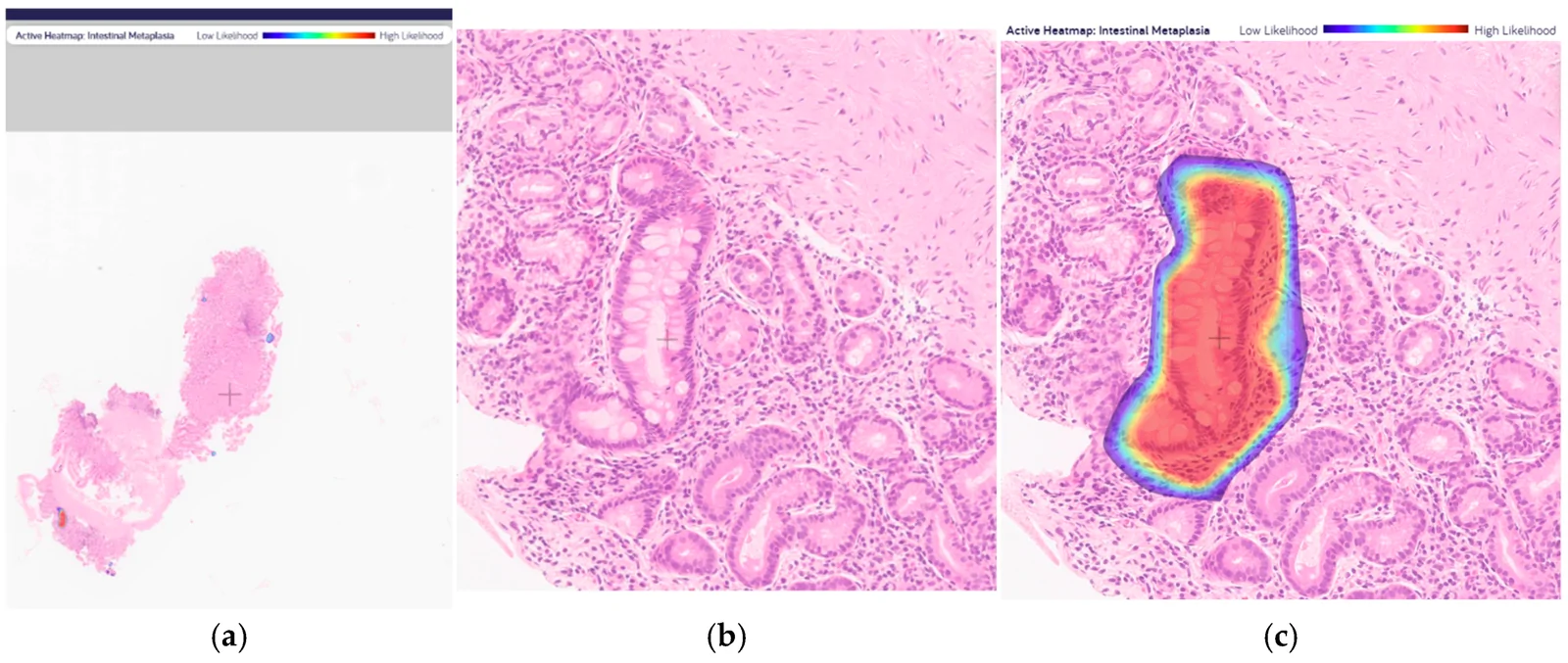
High-magnification AI heatmap capturing a focal region of gastric intestinal metaplasia missed during routine diagnosis: (**a**) digital H&E slide at 0.8× magnification of the specimen area; (**b**) digital H&E slide at 14× magnification of the region of interest; (**c**) digital H&E slide at 10× magnification of the region of interest with overlapping AI heatmap indicating high probability of intestinal metaplasia. The colour bar indicates predicted likelihood, ranging from blue (low likelihood) to red (high likelihood).

For gastric intestinal metaplasia (GIM), the platform achieved a sensitivity of 94.9% (95% CI: 88.6–97.8%), a specificity of 98.5% (95% CI: 97.4–99.2%), and an AUC of 0.967 (95% CI: 0.942–0.992) (Table 2). Beyond high baseline metrics, the AI detected 7 distinct cases of GIM that were missed in primary pathology reports (see example in Figure 4).

Unblinded secondary human review confirmed focal intestinal metaplasia in all 7 instances, leading to corrected diagnoses and initiation of risk-stratified surveillance.

### 3.4. Exploratory Evaluation of Low-Prevalence Lesions and Non-Neoplastic Conditions

Due to low epidemiological prevalence within the prospective cohort, standard diagnostic metrics could not be independently computed for neuroendocrine neoplasms (NEN) and low-grade B-cell lymphomas. These findings should therefore be interpreted as exploratory.

**Figure 5:**
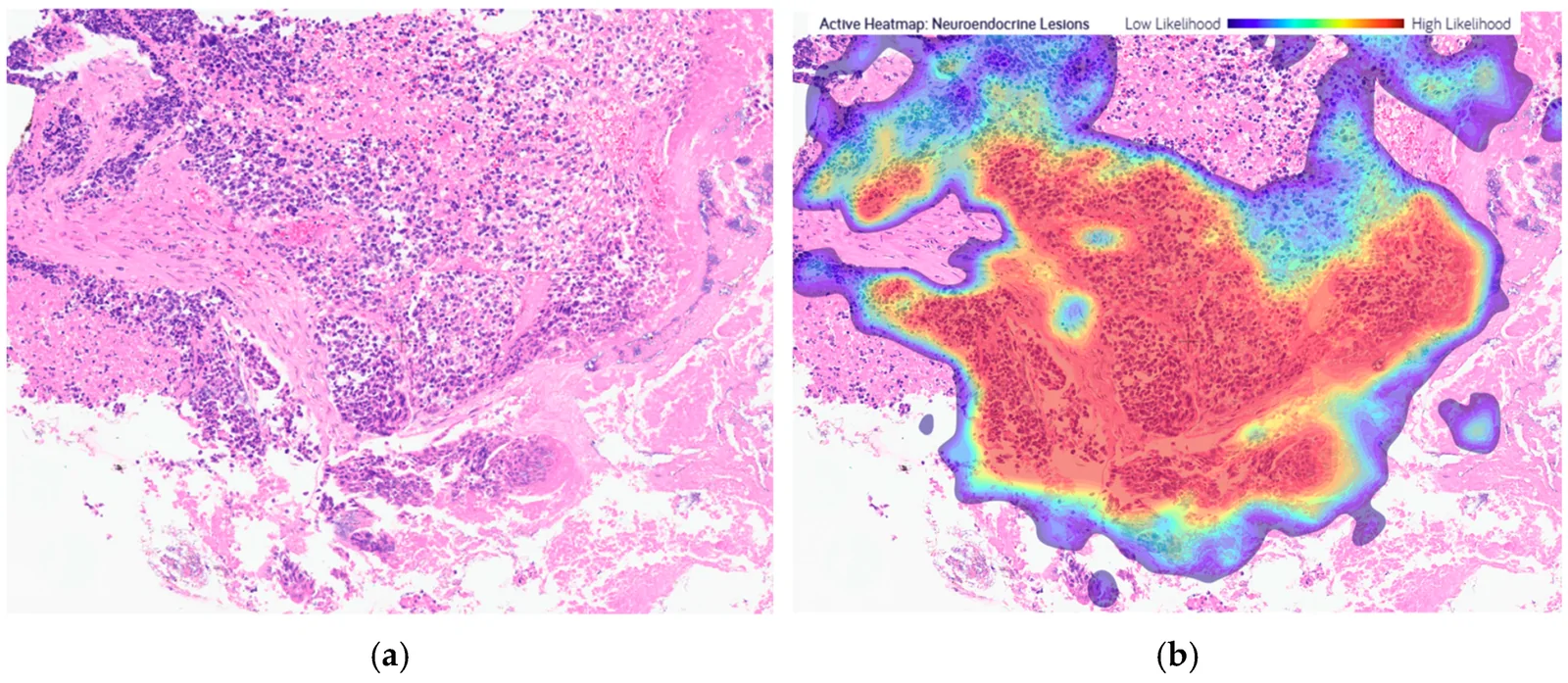
Automated detection and regional classification of a neuroendocrine neoplasm (NEN) case detected by AI: (**a**) digital H&E slide at 10× magnification of the region of interest; (**b**) digital H&E slide at 10× magnification of the region of interest with overlapping AI heatmap indicating high probability of neuroendocrine neoplasm (NEN). The colour bar indicates predicted likelihood, ranging from blue (low likelihood) to red (high likelihood).

Operationally, the platform successfully triaged 100% (*n* = 2) of NEN cases via primary automated alerts (example in Figure 5). For low-grade lymphomas, the platform flagged 75% of cases (*n* = 3/4). A single highly complex case involving a recurrence of gastric mucosa-associated lymphoid tissue (MALT) lymphoma failed to trigger the specific primary lymphoma classification alert. However, because the algorithm applies overlapping morphological risk heatmaps to detect generalised structural tissue atypia, the system flagged the region as highly suspicious for malignancy (93.0% structural anomaly score). The displayed heatmap overlay successfully directed the pathologist to the atypical lymphoid infiltrates, preventing a false negative at the workflow level.

Beyond neoplastic conditions, the platform also highlighted non-neoplastic pathologies, including gastritis, glandular atrophy, and hyperplastic/fundic gland polyps.

**Figure 6:**
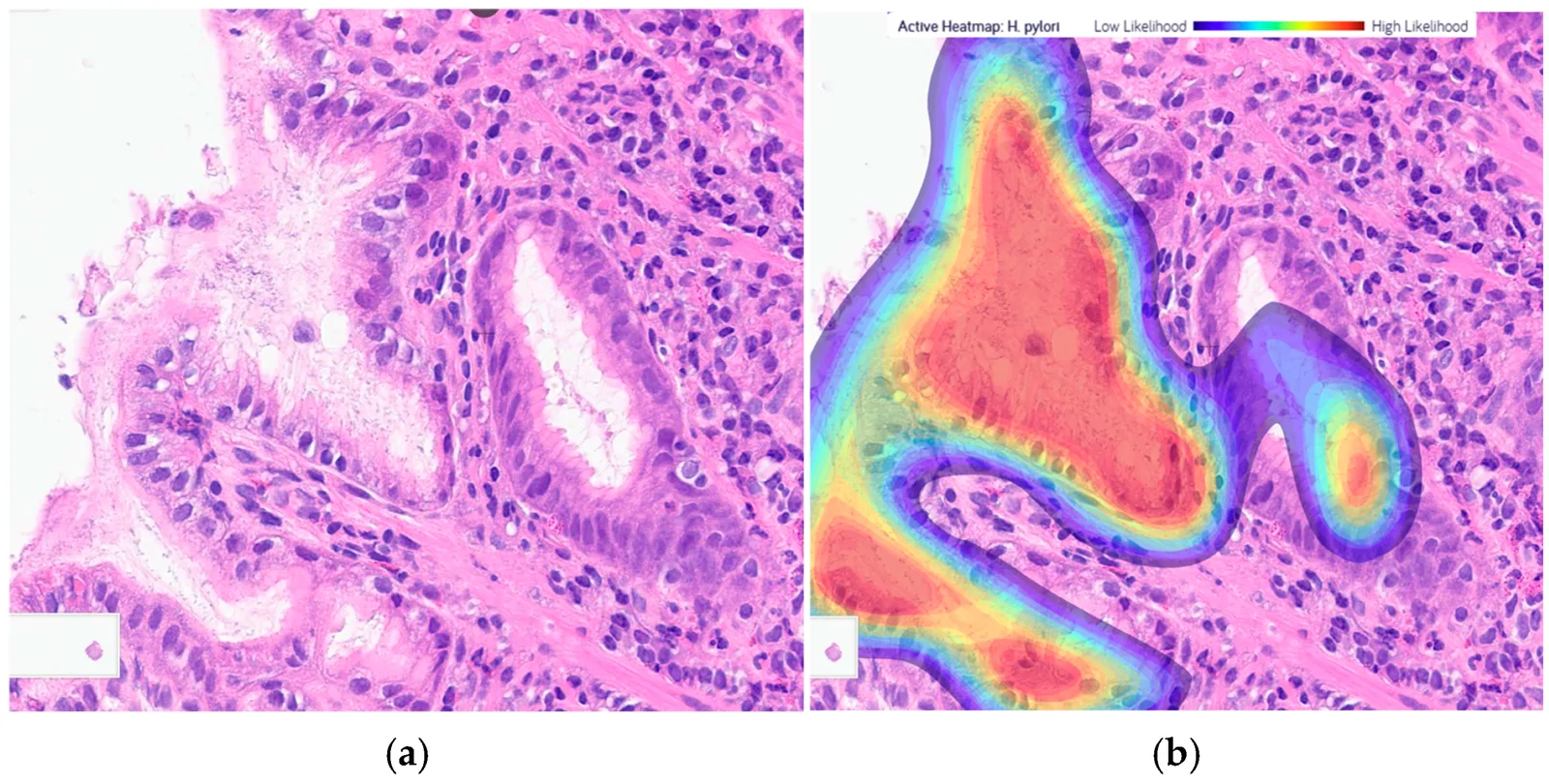
High-probability micro-regions flagged by the AI application to guide the identification of *Helicobacter pylori*: (**a**) digital H&E slide at 40× magnification of the region of interest; (**b**) digital H&E slide at 40× magnification of the region of interest with overlapping AI heatmap indicating high probability of *Helicobacter pylori*. The colour bar indicates predicted likelihood, ranging from blue (low likelihood) to red (high likelihood).

The AI also highlighted localised micro-regions suspicious for *Helicobacter pylori* infection (example in Figure 6), triggering visual cues that successfully prompted reflex IHC staining to confirm the presence of the pathogen in multiple routine cases. These observations are exploratory and do not establish diagnostic performance for these conditions.

### 3.5. Simulated Operational Workflow and Turnaround Time Impact

To determine the platform’s potential utility as an automated workflow orchestrator, the prospective testing dataset was post-analytically cross-tabulated against the original clinical triage pathways. This simulation evaluated the AI platform’s capacity to dynamically sort worklists by condition severity, up-stratifying high-risk cases regardless of their original designation as urgent or routine.

Across the multi-centre evaluation (*n* = 848), 149 total cases presented with verified clinical findings (97 urgent, 52 routine). The system demonstrated a 100.0% simulated triage safety rate for malignant disease, successfully identifying and prioritising all 34 cancer cases in the urgent pathway and all 6 cancer cases hidden within the routine backlog. Across the entire cohort, 23 of 848 cases (2.7%) generated false-positive cancer triage alerts (calculated as the number of false-positive cancer alerts divided by the total number of cases processed).

Under standard of care (SoC), routine cases experienced historical median turnaround times (TAT) of up to 26.0 working days (site maximum median) prior to reporting. In the simulated AI workflow, these misclassified routine malignancies were flagged for immediate prioritisation on Day 1 (immediately upon digitisation and processing) of the reading queue. The site-by-site breakdown of patient turnaround times is reported in Appendix B, Table A5.

The algorithm displayed comparable utility in flagging high-risk, non-malignant precursor conditions (*n* = 109). Within the urgent pathway, the platform successfully captured 96.8% of the 63 clinically significant non-cancer cases (*n* = 61/63). Within the routine pathway, it correctly isolated 97.8% of the 46 non-cancer cases (*n* = 45/46). Overall, the platform correctly triaged 97.2% (*n* = 106/109) of all non-cancer findings across both pathways (Table 3).

Crucially, the simulated triage did not compromise operational throughput. False-positive triage alerts occurred in 4.9% of all cases in the urgent pathway (*n* = 24/487) and 4.2% in the routine pathway (*n* = 15/361), corresponding to 4.6% of all cases in the total cohort (*n* = 39/848; Table 3). These false positive alert rates were calculated by dividing the number of false-positive triage alerts by the total number of cases processed in each pathway. These findings suggest that the platform could support automated worklist prioritisation while limiting the proportion of cases generating false alerts; however, the effects on pathologist workload and alert fatigue require prospective evaluation.

## 4. Discussion

The prospective, multi-centre evaluation of the Ibex Gastric AI platform across NHS Wales demonstrates a clinician-led real-world implementation in digital pathology, evaluating an artificial intelligence solution to pre-screen and triage consecutive gastric biopsies in near-real-time. By analysing a prospective cohort of 848 gastric biopsy cases across three major NHS Health Boards (BCUHB, HDUHB, and SBUHB), this evaluation addresses critical diagnostic backlog pressures and clinical safety requirements under routine, real-world operational conditions. The platform demonstrated high diagnostic accuracy across the evaluated pathological categories. Most notably, the AI platform achieved 100% sensitivity and 100% negative predictive value (NPV) for the detection of gastric malignancies (including adenocarcinoma, high-grade dysplasia, and high-grade lymphoma) and low-grade dysplasia/adenomas. These findings indicate strong detection performance in the evaluated cohort, although the wide confidence intervals reflect the low real-world prevalence of positive cases, particularly for low-grade dysplasia/adenomas.

For the other non-malignant precursor lesions, the AI platform showed high sensitivity for gastric intestinal metaplasia (GIM; 94.9%), with specificity above 98%. Eight primary clinical findings missed during routine human reporting, comprising one gastric adenocarcinoma and seven focal GIM cases, were identified by AI and confirmed on secondary review. Given the role of GIM in the Correa carcinogenesis pathway and its implications for surveillance and risk stratification, improved detection of focal metaplasia may enhance the consistency and safety of downstream clinical management. These results support the potential of AI to reduce diagnostic delay, strengthen early detection, and improve operational efficiency within high-volume histopathology services.

The operational data analysis highlighted significant structural bottlenecks within standard chronological workflows, wherein routine diagnostic pathways demonstrated historical baseline medians ranging from 14.0 to 26.0 working days (standard deviations reaching up to ±22.5 working days, with upper operational thresholds [mean + standard deviation] extending to 54 working days) prior to final sign-off. The most clinically important observation was the identification of malignant disease among cases originally assigned to the routine pathway. In conventional workflows, triage is frequently informed by endoscopic appearance, referral information, and clinical documentation. Although these inputs are valuable, they do not fully characterise the histological risk of every specimen [23]. In this cohort, six cancers were present among cases initially classified as routine, and the AI-based triage simulation identified all six. The historical turnaround time for this subgroup averaged up to 26 working days, compared with a near-real-time slide-level risk scoring immediately following digitisation that could automatically flag high-risk cases at the pipeline’s ingress for immediate prioritisation on Day 1 of the reading queue. This capability would allow health boards to dynamically prioritise diagnostic worklists, effectively transitioning pathology operations from a reactive, chronological paradigm to a proactive, risk-stratified framework. Importantly, this prioritisation was achieved with false-positive triage alerts in 4.6% of all processed cases, suggesting that relatively few cases would generate false alerts requiring additional review.

For the NHS, such a model could translate into earlier diagnosis and treatment initiation for high-risk patients, more efficient use of pathologist time, and reduced clinical risk from delayed or missed malignancies. Over time, these gains may support compliance with cancer-waiting-time targets, reduce amended reports and medico-legal exposure, and improve patient experience by shortening the diagnostic journey.

Beyond workflow optimisation, the AI platform demonstrated its clinical utility as an objective, parallel safety net as an addition to the standard of care review that enhanced the diagnostic accuracy of reporting pathologists. This synergistic relationship between human expertise and AI precision was demonstrated by the detection of a primary gastric adenocarcinoma in a case initially diagnosed as a benign gastric ulcer. Rather than replacing the physician’s judgement, the AI platform heatmap overlays prompted a secondary review that resulted in an updated final diagnosis after confirmation of cytokeratin-positive (CK+) cells. Thus, the AI platform could provide pathologists with an added layer of diagnostic confidence, ensuring that subtle malignancies are captured early and immediate oncological care is initiated. The distinction between algorithmic performance and workflow-level safety is important. A lesion may fail to receive a lesion-specific label, yet still generate a broader abnormality or malignancy finding. This was observed in the recurrent gastric MALT lymphoma case, in which the specific low-grade lymphoma heatmap did not achieve high likelihood, but did receive a high-likelihood score for carcinoma, directing the pathologist to the atypical lymphoid infiltrate. Such findings suggest that a layered system comprising lesion-specific classifiers, quality-control modules, and general abnormality detection may be more clinically useful than narrow categorical outputs such as binary classifiers, because integrated architectures can maintain safety when rare or atypical entities fall outside a narrow training distribution while still providing high-performance detection.

Furthermore, the platform showed potential as a multi-purpose diagnostic aid for gastric biopsy assessment, generating alerts for rare lesions such as neuroendocrine neoplasms and highlighting localised regions suspicious for *H. pylori*, which prompted reflex staining in routine cases.

To fully build upon this successful evaluation, certain limitations must be addressed in subsequent studies. While the multi-centre design across three major Health Boards captured real-world operational heterogeneity, the prospective testing window was limited to three months (between March and June 2025). Consequently, several study limitations should be noted. First, certain diagnostic categories contained small numbers of positive cases, particularly low-grade dysplasia and rare neoplasms, resulting in wider confidence intervals for sensitivity estimates for these categories. Second, the reference standard was based on routine clinical diagnoses, with discrepant cases undergoing unblinded secondary adjudication through discussion between at least two expert pathologists and ancillary tests. Third, external generalisability remains constrained because, despite differences in tissue collection, processing and laboratory procedures, all three participating Health Boards operate within the same public healthcare system (NHS Wales) and use a single model slide scanner platform (Leica Aperio GT 450), although different scanners are used at each hospital. Although the underlying AI algorithm was trained on international, multi-vendor datasets, prospective validation across diverse international healthcare systems and alternative slide scanner platforms is necessary to establish its broader generalisability. Finally, turnaround-time improvements were evaluated through post-analytic workflow simulation rather than a prospective interventional trial in which AI directly altered the live reporting order.

Long-term health economic and clinical outcome studies are required to quantify exactly how this accelerated triage impacts long-term survival rates and pathologist and patient satisfaction metrics within the health boards.

To conclude, this multi-centre evaluation demonstrated that the AI platform evaluated in the study provided a highly accurate, pathologist-led safety net and automated workflow triaging within public healthcare infrastructure. These findings suggest that AI-assisted gastric-biopsy review can improve detection of cancer and precursor lesions, prioritise high-risk cases within routine backlogs, and do so with low operational friction. For the NHS, this offers a scalable model to enhance diagnostic safety, reduce delays, and move from chronological processing toward risk-stratified, proactive pathology services.

## Figures and Tables

**Table 1:** Distribution of Routine and Urgent Cases Across Participating Sites.

Health Board/Site	Pathway	Uploaded Cases (*n*)	Final Cohort (*n*)
BCUHB	Urgent	249	220
	Routine	284	260
HDUHB	Urgent	102	80
	Routine	43	35
SBUHB	Urgent	216	187
	Routine	88	66
Total	Urgent	567	487
	Routine	415	361
	Total	982	848

**Table 2:** Diagnostic performance of the AI platform across the participating sites.

Pathological Category	Total Cases (*n*)	Sensitivity % (95% CI)	Specificity % (95% CI)	PPV % (95% CI)	NPV % (95% CI)	AUC (95% CI)	Detected by AI/Missed by Pathologist (*n*)
Gastric Malignancies	848	100.0% (91.2–100.0%)	97.2% (95.8–98.1%)	63.5% (51.1–74.3%)	100.0% (99.5–100.0%)	0.986 (0.960–1.000)	1
Low-Grade Dysplasia/Adenoma	842	100.0% (75.8–100.0%)	98.0% (96.7–98.7%)	41.4% (25.5–59.3%)	100.0% (99.5–100.0%)	0.990 (0.950–1.000)	0
Gastric Intestinal Metaplasia	842	94.9% (88.6–97.8%)	98.5% (97.4–99.2%)	89.4% (82.0–94.0%)	99.3% (98.4–99.7%)	0.967 (0.942–0.992)	7

**Table 3:** AI simulated Triage Performance and Baseline Timelines Across Pathways.

Baseline Pathway Designation	Total Cases (*n*)	Confirmed Malignancies (*n*)	Cancer Cases Correctly Triaged by AI (%)	Clinically Significant Non-Cancer Cases (*n*) *	Clinically Significant Non-Cancer Triaged by AI (%)	False-Positive Triage Alert Rate (%) **	Historical Baseline TAT: Median Working Days ***
Urgent Pathway	487	34	100.0%	63	96.8%	4.9%	9–15
Routine Pathway	361	6	100.0%	46	97.8%	4.2%	14–26
Total Cohort	848	40	100.0%	109	97.2%	4.6%	-

* clinically significant non-cancer cases include neuroendocrine lesions, low-grade dysplasia, adenoma, and intestinal metaplasia. ** false-positive triage alert rate was defined as AI-triggered alerts in cases without the corresponding clinically significant finding. Percentages were calculated as the number of false-positive triage alerts divided by the total number of cases processed in the corresponding pathway (FP/N_total_). *** the TAT range represents the minimum and maximum median values observed across the three participating NHS Health Boards following the exclusion of outliers (1.5 × IQR). A complete multi-centre breakdown is provided in Table A5.

## Data Availability

The clinical data generated and analysed during this evaluation are not publicly available due to patient privacy, confidentiality, and data protection requirements under the UK General Data Protection Regulation (UK GDPR) and applicable institutional policies. The participating NHS Health Boards retain control of and responsibility for the clinical data, with the AI platform provider acting as a data processor on behalf of the Health Boards in accordance with the applicable Data Protection Impact Assessment (DPIA) and data-processing arrangements. Access to the underlying clinical dataset is therefore restricted, and it cannot be made publicly available.

## Appendix A

### Diagnostic Performance Across Participating Sites

This supplementary section reports AI diagnostic performance across the three participating NHS Health Boards: Betsi Cadwaladr University Health Board (BCUHB, *n* = 480), Hywel Dda University Health Board (HDUHB, *n* = 115), and Swansea Bay University Health Board (SBUHB, *n* = 253). Full counts of true positives (TP), false positives (FP), true negatives (TN), and false negatives (FN) for each target condition are reported within the tables. Site-level performance is summarised using sensitivity, specificity, positive predictive value (PPV), and negative predictive value (NPV) at the predefined clinical triage threshold. However, the low prevalence of some target conditions at individual sites results in small numbers of positive cases and consequently wide confidence intervals, which may limit the precision and interpretability of site-specific estimates. Nevertheless, these results are reported to provide transparency and allow assessment of performance across the participating Health Boards. Confidence intervals for all reported proportions were calculated using the Wilson score method. AUC and its 95% confidence interval are reported for the aggregate cohort but not at the site level, as the small number of positive cases for some target conditions (e.g., 3–5 cases for low-grade dysplasia/adenoma) limits the precision and interpretability of site-specific estimates. Confidence intervals for site-level sensitivity, specificity, PPV, and NPV were calculated using the Wilson score method.

**Table A1:** Multi-Centre Aggregate Diagnostic Performance (Total Cohort).

Pathological Category	Total Cases (*n*)	TP	FP	FN	TN	Sensitivity % (95% CI)	Specificity % (95% CI)	PPV % (95% CI)	NPV % (95% CI)	AUC (95% CI)
Gastric Malignancies	848	40	23	0	785	100.0% (91.2–100.0%)	97.2% (95.8–98.1%)	63.5% (51.1–74.3%)	100.0% (99.5–100.0%)	0.986 (0.960–1.000)
Low-Grade Dysplasia/Adenoma	842	12	17	0	813	100.0% (75.8–100.0%)	98.0% (96.7–98.7%)	41.4% (25.5–59.3%)	100.0% (99.5–100.0%)	0.990 (0.950–1.000)
Gastric Intestinal Metaplasia	842	93	11	5	733	94.9% (88.6–97.8%)	98.5% (97.4–99.2%)	89.4% (82.0–94.0%)	99.3% (98.4–99.7%)	0.967 (0.942–0.992)

**Table A2:** Site-Level Diagnostic Performance: Betsi Cadwaladr University Health Board (BCUHB).

Pathological Category	Total Cases (*n*)	TP	FP	FN	TN	Sensitivity % (95% CI)	Specificity % (95% CI)	PPV % (95% CI)	NPV % (95% CI)
Gastric Malignancies	480	13	7	0	460	100.0% (77.2–100.0%)	98.5% (96.9–99.3%)	65.0% (43.3–81.9%)	100.0% (99.2–100.0%)
Low-Grade Dysplasia/Adenoma	479	5	8	0	466	100.0% (56.6–100.0%)	98.3% (96.7–99.1%)	38.5% (17.7–64.5%)	100.0% (99.2–100.0%)
Gastric Intestinal Metaplasia	479	53	8	1	417	98.1% (90.2–99.7%)	98.1% (96.3–99.0%)	86.9% (76.2–93.2%)	99.8% (98.7–100.0%)

**Table A3:** Site-Level Diagnostic Performance: Hywel Dda University Health Board (HDUHB).

Pathological Category	Total Cases (*n*)	TP	FP	FN	TN	Sensitivity % (95% CI)	Specificity % (95% CI)	PPV % (95% CI)	NPV % (95% CI)
Gastric Malignancies	115	14	6	0	95	100.0% (78.5–100.0%)	94.1% (87.6–97.2%)	70.0% (48.1–85.5%)	100.0% (96.1–100.0%)
Low-Grade Dysplasia/Adenoma	114	3	3	0	108	100.0% (43.9–100.0%)	97.3% (92.4–99.1%)	50.0% (18.8–81.2%)	100.0% (96.6–100.0%)
Gastric Intestinal Metaplasia	114	18	2	2	92	90.0% (69.9–97.2%)	97.9% (92.6–99.4%)	90.0% (69.9–97.2%)	97.9% (92.6–99.4%)

**Table A4:** Site-Level Diagnostic Performance: Swansea Bay University Health Board (SBUHB).

Pathological Category	Total Cases (*n*)	TP	FP	FN	TN	Sensitivity % (95% CI)	Specificity % (95% CI)	PPV % (95% CI)	NPV % (95% CI)
Gastric Malignancies	253	13	10	0	230	100.0% (77.2–100.0%)	95.8% (92.5–97.7%)	56.5% (36.8–74.4%)	100.0% (98.4–100.0%)
Low-Grade Dysplasia/Adenoma	249	4	6	0	239	100.0% (51.0–100.0%)	97.6% (94.8–98.9%)	40.0% (16.8–68.7%)	100.0% (98.4–100.0%)
Gastric Intestinal Metaplasia	249	22	1	2	224	91.7% (74.2–97.7%)	99.6% (97.5–99.9%)	95.7% (79.0–99.2%)	99.1% (96.8–99.8%)

## Appendix B. Operational Baseline Data

To establish a realistic baseline for Standard of Care (SoC) laboratory throughput and prevent artificial distortion from severe operational anomalies (e.g., long-term patient-requested holds or administrative LIMS closing delays), raw turnaround times were preprocessed using the Interquartile Range (1.5 × “IQR”) outlier removal rule:

1. Interquartile Range Calculation: For each health board and pathway cohort, the first quartile (Q_1, 25^“th” percentile) and third quartile (Q_3, 75^“th” percentile) of turnaround times were determined.
2. Interquartile Range (IQR): Defined as IQR = Q_3 − Q_1.
3. Threshold Boundaries: Lower boundary = Q_1 − 1.5 (IQR); Upper boundary = Q_3 + 1.5 (IQR).
4. Exclusion: Data points lying outside these boundaries were excluded from the final descriptive statistics (mean, standard deviation, and median calculations).

Working days were calculated strictly as weekdays (Monday through Friday), excluding UK statutory bank holidays. Patient turnaround times (TAT), calculated from the date of specimen collection to report authorisation (sign-off), are presented in Table A5 for completeness.

**Table A5:** Baseline Turnaround Time (TAT) and Throughput Metrics Across Participating NHS Health Boards (Working Days).

Metric/Parameter	Betsi Cadwaladr (BCUHB)		Swansea Bay (SBUHB)		Hywel Dda (HDUHB)	
	Routine	Urgent	Routine	Urgent	Routine	Urgent
Total Patients (*n*)	595	295	119	298	290	66
Evaluation Period (Days)	142	146	146	147	155	153
Evaluation Period (Weeks)	20	21	21	21	22	22
Patients per week (*n*)	29.4	14.1	5.7	14.2	13.1	3
TAT-Average (working days)	24.5	15.3	31.7	17.2	17.1	10.3
TAT-Std Dev (working days)	8.2	8.0	22.5	8.6	11.5	5.6
Variation (%)	33.6%	51.9%	71.1%	50.3%	67.0%	54.1%
TAT-Median (working days)	26.0	13.5	24.0	15.0	14.0	9.0
Reported ≤15 working days (%)	15.6%	58.3%	26.9%	53.3%	58.7%	88.3%
Reported ≤30 working days (%)	71.8%	87.1%	63.9%	89.8%	82.6%	100.0%
Reported ≤45 working days (%)	99.8%	98.6%	73.9%	100.0%	97.8%	100.0%
Reported ≤60 working days (%)	100.0%	100.0%	84.0%	100.0%	100.0%	100.0%
